# Epigenetics and Attention-Deficit/Hyperactivity Disorder: New Perspectives?

**DOI:** 10.3389/fpsyt.2020.00579

**Published:** 2020-06-17

**Authors:** Bojan Mirkovic, Abdeslam Chagraoui, Priscille Gerardin, David Cohen

**Affiliations:** ^1^Department of Child and Adolescent Psychiatry, CH Le Rouvray, Rouen University Hospital, Rouen, France; ^2^Université Paris-Saclay, UVSQ, INSERM, Centre for Research in Epidemiology and Population Health (CESP), Villejuif, France; ^3^Neuronal and Neuroendocrine Differentiation and Communication Laboratory, Institute for Research and Innovation in Biomedicine of Normandy (IRIB), Department of Medical Biochemistry, Rouen University Hospital, Rouen, France; ^4^Department of Child and Adolescent Psychiatry, AP-HP, Groupe Hospitalier Pitié-Salpêtrière, Paris, France; ^5^GRC-15, Approche dimensionnelle des épisodes psychotiques de l'enfant et de l'adolescent, Faculté de Médecine, UPMC, Sorbonne Université, Paris, France; ^6^CNRS UMR 7222 “Institut des Systèmes Intelligents et Robotiques”, Sorbonne Université, Paris, France

**Keywords:** epigenetics, attention deficit/hyperactivity disorder, methylation, microRNA, biomarkers

## Introduction

Attention deficit/hyperactivity disorder (ADHD) is a common neurodevelopmental disorder with a heavy burden for individuals, families, and society (1). Although ADHD runs within families, modeling causality in ADHD is a complex endeavor, and several models have been proposed combining both genetic and environmental factors (2). Statistical heritability does not correspond to heredity, and it does not describe the biological process by which a disorder emerges. Heritability also includes Gene x Gene or Gene x Environment interactions, and, transgenerational or developmental effect of epigenetic traces (3). DNA based transmission is not the exclusive mode of inheritance in humans, as at least three other modes—epigenetic-, behavioral-, and symbol-based—have been described (4). Like other inheritance systems, the symbolic system (e.g., the language system) not only allows humans to transmit information to others but also allows humans to communicate with themselves: symbolic communication is a way of thinking (4).Although how and when environmental factors impact the brain of ADHD individuals remains unclear in most cases, it is likely that several mechanisms occur: (i) direct toxic impact; (ii) genetic modulation of a given environmental factor; (iii) environmental modulation of a given genetic effect; and/or (iv) epigenetics modifications through both early and transgenerational constraints (5). In recent years, the epigenetic perspective has aroused growing interest to improve understanding of ADHD and identify potential biomarkers.

### What Is Epigenetics?

**Figure 1:**
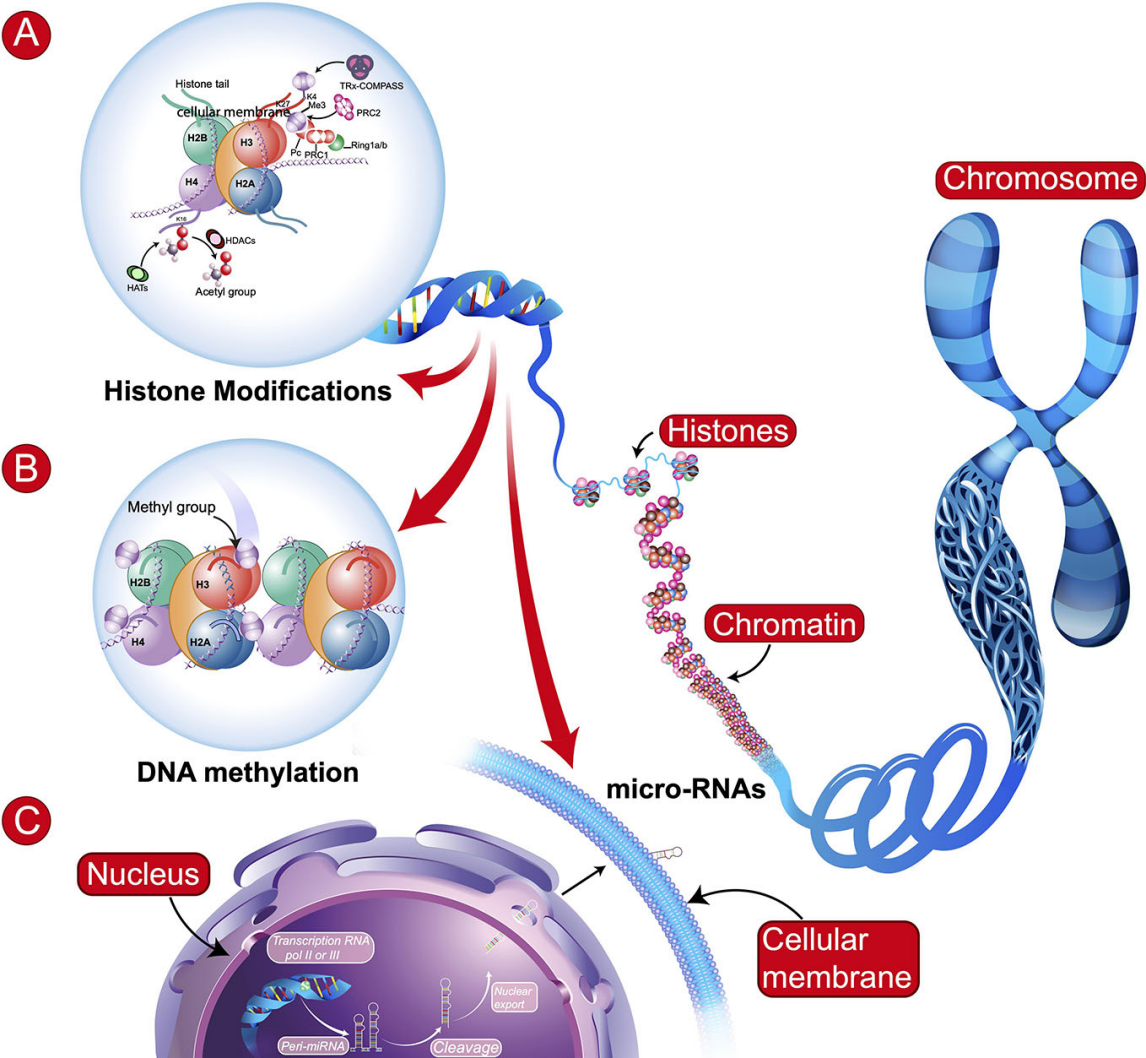
Schematic representation of main epigenetic mechanisms. **(A)** Histone Modifications: These modifications involve: histone acetyltransferases (HATs), histone deacetylases (HDACs), or histone methyltransferases [e.g., tri-methylation the 4th lysine residue of the histone protein (H3K4Me3) involving Polycomb (PRC1 and PRC2), Trithorax (TRx), and COMPASS (complex proteins associated with SET1)]. **(B)** DNA Methylation: transfer of a methyl group (CH3) onto the C5 position of the cytosine to form 5-methylcytosine. In (A, B): H2B, H3, H4, H2A = Core histone. **(C)** microRNAs (regulatory RNAs) can affect alternative splicing and protein expression. Illustration of biosynthesis: transcription of miRNA gene by RNA polymerase II or III to form primary miRNA (Pri-miRNA), which are then cleaved and some may be circulating.

The definition of epigenetics has evolved since the seminal works of Conrad H. Waddington and Victor H. Denenberg in the sixties. According to Bird (6), epigenetics is the study of the inheritance of alternative chromatin states in the absence of changes in the DNA sequence. The best described epigenetic processes are the addition of methyl groups (CH3) to DNA (methylation) and posttranslational modifications to histone proteins, such as methylation, phosphorylation or acetylation (**Figure 1**). More recently, other epigenetic mechanisms have been described, such as hydroxymethylation of DNA (7) investigated in neurodevelopmental disorders or dopaminylation (H3Q5dop) involved in cocaine-induced transcriptional plasticity in the midbrain (8).

Epigenetic processes are involved in cell differentiation and in the main neurobiological and cognitive processes in the brain, and represent both a potential index of pathophysiological mechanisms and a possible source of new biomarkers. Epigenetic processes influence gene expression by acting dynamically on the compacting of chromatin and, therefore, on the accessibility of the transcriptional machinery. Unlike relatively stable DNA (genome) sequences, the epigenome is altered in a dynamic and sometimes reversible manner by exposure to many intrinsic or extrinsic environmental factors. Moreover, some changes induced in the early stages of life can remain silent until their biological influence is triggered later in life. Recently, a study examined the association between various types of childhood adversity and genome-wide DNA methylation in two large female population-based cohorts and showed nine differentially methylated genomic regions (DMRs) (9). Moreover, the authors showed that only one DMR was associated with a measure of cumulative adversity, while the other eight regions were associated with specific types of adversity, namely parental mental illness, physical illness, or death. This suggests that some parts of the methylome are associated with distinct negative childhood experiences, while other sites reflect more general exposure to adversity.

The inheritance of epigenetic markers during meiosis and mitosis is complex, particularly for histone modifications (chromatin heritability) because nucleosomes do not have a duplication system based on a DNA model (7). The hierarchical and three-dimensional organization of the genome shows that epigenetic inheritance involves several layers. The successive layers bring stability but are reversible. This allows considerable plasticity and ultimately less deterministic consequences. The variation in DNA sequences adds a degree of complexity to the understanding of epigenetic mechanisms: the variation in the chromatin state can influence the binding of a transcription factor, and the polymorphism of the DNA sequence can influence the chromatin states (7).

### How Important Is the Environment in ADHD?

Environmental risk factors are numerous in understanding ADHD causality (10). Epigenetics offers a possible mechanism for the interactions between environment, intrinsic signals, regulation of gene activities, and the genes themselves. Classifying environmental factors requires distinguishing risk factors from protective factors and moderators from mediators. Also, it implies distinguishing toxins, micro- and macro-level factors according to their respective major impact level (subject's distance) (5). Toxins usually impact the brain during the perinatal period (smoking, alcohol). Environmental variables may be passed on *via* intermediary variables; for example, the correlation between cigarette consumption during pregnancy and birth weight/intrauterine growth retardation. It may also pass on through genetic modulations (e.g., polymorphism of dopamine transporter) (3). Finally, transgenerational effects have been described as the exposure of grandparents to certain pollutants, which leads to a 30% increase in the risk of ADHD in their grandchildren (11). However, these studies remain rare, and their numerous methodological limitations mean that great caution should be used in interpreting the results. The second type of environmental factors includes individual and familial factors, known as microenvironments. These include physical or sexual abuse, lack of family discipline, use of extreme punishment, poverty, minority status, an urban environment, a family history of psychiatric disorders, and isolated parents. Macro-environmental factors are more difficult to demonstrate with clear evidence because they require cross-cultural or longitudinal studies with a large number of participants to control for confounding factors (2). Thus, the role of environment in ADHD is essential. Given the complexity of the links between environment, genetics, and epigenetics, such integration work must necessarily include genetically informed environmental studies and epigenetically informed genetic studies.

### DNA Methylation Changes in ADHD

Historically, a high level of DNA methylation has been associated with repression of gene expression, but the most recent data have shown that this association can vary depending on numerous factors including the location of the methylation in the gene sequence (12). DNA methylation can be mapped either at the global level (overall level of methylcytosine -5mC- in the genome, expressed as a percentage) or at the site-specific level (candidate gene association studies) or genome-wide levels at single nucleotide resolution.

It is difficult to study epigenetic effects on human behaviour because epigenetic changes vary widely from one tissue to another. In the brain alone, DNA methylation can vary considerably from one region to another or from one system to another. However, there are also cross-tissue correlation patterns of individual-specific epigenetic marks so that DNA methylation studies of peripheral tissue DNA in psychiatry are generally considered informative (13). The existence of age-linked changes of methylation profiles (the methylome-based clock) constitutes an additional level of complexity.

The significance of DNA methylation in ADHD stems mainly from studies (on humans and animals) which have shown aberrant methylation profiles in the event of exposure to risk factors strongly associated with ADHD such as lead, maternal smoking or low birth weight (14).

Overall, studies on the association between DNA methylation and ADHD symptoms based on the candidate gene approach (mainly dopaminergic genes) are rare, with small numbers of subjects, and have shown disappointing and often contradictory results, comparable to SNP association studies (15). The most robust results come from studies at the epigenome level, but unfortunately, these studies are rare and mainly cross-sectional, making it impossible to examine whether altered DNA methylation models are a risk factor and/or a consequence of ADHD or to establish the stability of the associations over time.

The first Epigenome-Wide Association Studies (EWAS) identified altered methylation in new genes that were not previously associated with ADHD such as MYT1L (encodes a transcription factors whose expression, thus far, has been found only in neuronal tissues) and VIPR2 (16). VIPR2 encodes a receptor for vasoactive intestinal peptide, and is expressed throughout the central and peripheral nervous system. It is interesting to note that an alteration in methylation at the VIPR2 gene was also identified in a study of 14 pairs of monozygotic twins discordant for ADHD (17) and in a study analyzing the associations between methylation profile, early childhood malnutrition, and ADHD (18).

The only prospective study included 817 subjects and assessed DNA methylation at birth and age 7 years, and trajectories of ADHD symptoms (7–15 years) (19). The authors identified 13 genomic locations where the level of DNA methylation was significantly predictive of the trajectories of ADHD symptoms between 7 and 15 years of age. Among the loci identified was ST3GAL3, one of the 12 significant loci of the largest GWAS available to date for ADHD (20). ST3GAL3 (encodes the Golgi enzyme β-galactoside-α2,3-sialyltransferase-III) mutations seem to be implicated in the development of higher cognitive functions.

However, other EWAS have reported disappointing results. For example, the first EWAS meta-analysis (21), based on three population-based adult cohorts (N = 4.689) found different methylated positions in one cohort, but the meta-analysis of all the cohorts failed to detect any significant difference.

In order to better take into account the complexity of gene regulation and the impact of the environment, so-called second generation studies incorporating epigenetics and genetics have been developed. A large integrated epigenetic/genetic study of 391 children with ADHD reported an association between ADHD and DNA methylation levels at sites annotated to VIPR2 and several novel differentially methylated positions, although none of them were genome-wide significant (22).

Several limitations have been suggested to explain the discordant results. First, the HumanMethylation450K matrix (the most widely used one) captures only about 1.7% of all CpGs in the genome. The most recent study available used a Beadchip kit (Infinium Methylation EPIC) to quantitatively interrogate 850Kmethylation sites across the genome at single-nucleotide resolution, but this failed to yield any significant results after correction for multiple tests.

Second, a certain level of methylation without CpGs and the presence of the oxidized form of 5-methylcytosine (5mC) and 5-hydroxymethylcytosine (5hmC) have been shown, but most common DNA methylation methods do not allow this distinction to be made. Third, cellular heterogeneity within the samples or the intrinsic variability of the methylation profiles are not considered.

Progress in the field of genome engineering has enabled more precise site-specific intervention on methylation processes versus the global approach at the genome level. Site-specific methylome editing will become a key technique for studying 5mC function/therapy. Indeed, it seems that DNA methylation can be complementary if it involves both alleles or non-complementary if it affects only one allele (allele-specific methylation [ASM]), especially since the ASM regions seem particularly sensitive to the effects of environment. Combining data from GWAS and from two post-mortem studies on ASM variants, a recent study examined the possible association between SNPs that show ASM in multiple brain regions and ADHD (23). The authors identified three genes, ARTN, C2orf82, and PIDD1, previously implicated in psychiatric disorders, and eight ASM tagSNPs, as significantly associated with ADHD. The identified risk variants had an impact on DNA methylation levels in the promoter regions, and were inversely correlated with expression of the corresponding genes in the brain.

Ultimately, the methylation differences associated with ADHD reported so far are generally small. Future work should take into account not only the limits discussed below but also the effects of gender or medication and the potential moderators of the stability and/or change of methylation over time. Moreover, it is likely that several methylation sites can interact together to contribute to phenotypic variations. Thus, as with genetic variations and polygenic scores, combinations of methylation sites could be used to develop potential predictive signatures.

### MicroRNAs in ADHD

MicroRNAs (miRNAs) have implications in post-transcriptional regulation and are particularly abundant in the nervous system, where they exert considerable influence on development and neuroplasticity (24). The deregulation of miRNAs could be involved in the pathophysiology of various neuropsychiatric disorders including ADHD. While there is no consensus on how the expression of these molecules is regulated, DNA methylation seems to be key because the synthesis of miRNAs can be regulated by DNA methylation and vice versa (25). The available studies on the role of miRNAs in the pathophysiology of ADHD are mainly of two types: (i) investigation of the role of gene polymorphisms (SNP) within miRNAs or miRNA target sites and (ii) investigation of circulating miRNAs (cimiRNAs).

In the largest association study focused on 134 miRNAs in 754 subjects with ADHD and 766 controls (26), the authors found a significant association between the miR-34b/c locus and ADHD, and an overexpression of the miR-34c-3p white blood cells of ADHD subjects. In addition, having tested the effect of SNPs within the ADHD-associated region on gene expression, they found that rs4938923 in the promoter of the pri-miR-34b/c is a tag for cis-expression quantitative trait loci for both miR-34b and miR-34c, and that it affects the expression levels of 681 transcripts in trans, including genes that had already been implicated in ADHD.

Circulating microRNAs are stable and easily quantifiable. Pathological processes in the central nervous system can be reflected in peripheral tissues (17). Many circulating miRNAs (18a-5p, 22-3p, 24-3p, 106b-5p, 107, 155a-5p, let-7d) have been associated with ADHD, including *via* next-generation sequencing (27–29).

Although profiling miRNAs as a molecular signature of ADHD is an interesting avenue to explore, there have not, to date, been enough studies, and many uncertainties remain. The available studies have not taken sufficient account of the fact that miRNAs can overlap in different psychiatric disorders, as does the distinction between adult and child ADHD. Moreover, the studies have mainly involved a single miRNA rather than several, which does not seem to correspond to the etiological complexity of ADHD. Indeed, the synthesis of miRNAs can be regulated by DNA methylation and vice versa, adding a new layer of complexity to the post-transcriptional regulation of gene expression (30). Finally, although the alteration of miRNAs in the CNS is also reflected in peripheral tissues (31), one study has recently found that the levels of microRNA 134 in plasma were not correlated with the levels in the CNS (24); future studies are needed to see if it is possible to use the expression of peripheral miRNAs as a proxy for miRNA activity in the brain.

## Concluding Remarks

We are aware that a major limitation of epigenetic markers is that their variation may be secondary to the development of a trait or may be a marker of environmental exposures. However, the value of a biomarker is defined by its ability to predict the state or the course of the disease, including its response to treatment, regardless of its value for understanding the pathophysiology. If the findings on epigenetic associations with common environmental exposures are confirmed in ADHD, this could have important consequences for public health policies. Primary prevention actions could be undertaken to reduce certain common environmental exposures, which would eventually reduce the incidence of ADHD and other neurodevelopmental disorders. The provision of easily quantifiable biomarkers could help clinicians deal with diagnostic process in complex cases by identifying vulnerable subjects and those responsive to treatment.

## Author Contributions

All the authors contributed to the conceptualization and the drafting of the paper and they critically reviewed the manuscript.

## Conflict of Interest

The authors declare that the research was conducted in the absence of any commercial or financial relationships that could be construed as a potential conflict of interest.
